# Changes in melatonin and sex steroid hormone production among men as a result of rotating night shift work – the HORMONIT study

**DOI:** 10.5271/sjweh.3991

**Published:** 2021-12-30

**Authors:** Barbara N Harding, Gemma Castaño-Vinyals, Anna Palomar-Cros, Kyriaki Papantoniou, Ana Espinosa, Debra J Skene, Benita Middleton, Alex Gomez-Gomez, José Maria Navarrete, Patricia Such, Antonio Torrejón, Manolis Kogevinas, Oscar J Pozo

**Affiliations:** 1 Barcelona Institute of Global Health (ISGlobal), Barcelona, Spain; 2 Universitat Pompeu Fabra, Barcelona, Spain; 3 Department of Epidemiology, Center of Public Health, Medical University of Vienna, Vienna, Austria; 4 University of Surrey, Guildford, United Kingdom; 5 Hospital del Mar Medical Research Institute (IMIM), Barcelona, Spain; 6 Seat, Barcelona, Spain; 7 Consortium for Biomedical Research in Epidemiology & Public Health (CIBER en Epidemiología y Salud Pública-CIBERESP), Madrid, Spain

**Keywords:** circadian, night work, rotating shift work, shift worker

## Abstract

**Objective:**

Data from real world settings on circadian disruption and subsequent hormone-related changes may explain the higher risk of hormone-dependent cancers among night shift workers.The present study examines the melatonin and sex steroid hormone levels among night shift workers.

**Methods:**

We included 44 male, rotating shift workers from a car factory in Spain, sampled both at the end of a 3-week night shift (22:00–06:00 hrs) and a 3-week early morning shift (06:00–14:00 hrs). Participants collected all urine voids over 24-hours during each shift. Urinary concentrations of sex steroid hormones (estrogens, androgens and progestogens) and 6-sulfatoxymelatonin (aMT6s, major melatonin metabolite) were determined. Individual cosinor analysis was used to derive the acrophase (peak time) and area under the curve (total production). Linear mixed models examined intraindividual associations between night shift work and log-transformed 24-hour peak time and total production of hormones compared to early morning shift work.

**Results:**

The acrophase was delayed during the night shift for aMT6s [geometric mean difference (GMD) 7.53 hrs, 95% confidence interval (CI) 4.46–10.60], androgens (eg, testosterone: GMD 6.83 hrs, 95% CI 0.34–13.32) and progestogens (eg, 17-hydroxyprogesterone: GMD 4.54 hrs, 95% CI 2.92–6.16) compared to the early morning shift. We found a higher production of adrenal androgen 11-oxoandrosterone/11-oxoetiocholanolone [geometric mean ratio (GMR) 1.43, 95% CI 1.12–1.81], and a lower production of adrenal progestogen 16-cysteinylprogesterone (GMR 0.79, 95% CI 0.67–0.93) during the night shift compared to the early morning shift levels.

**Conclusions:**

Night shift work was associated with melatonin and sex hormone-related changes in timing and total production, providing insight into the mechanistic path for its association with hormone-dependent cancer.

Internal biological processes are regulated by a central “clock” in the suprachiasmatic nuclei in the brain and peripheral clocks in virtually all tissues. These clocks dictate the rhythm of many human biological processes, including hormone production (1, 2). These rhythms can be influenced by external factors, such as light, a major environmental cue leading to the synchronization of endogenous circadian rhythms to the 24-hour day (3).

With approximately 19% of the European population working atypical hours including work during the night (4), and the expansion of human activities over the 24-hour day, examining the impact of circadian misalignment on health is an important priority. In 2007 and again in 2019, the World Health Organization’s International Agency for Research on Cancer (IARC) determined that shift work is “probably carcinogenic to humans,” (Group 2A) based on sufficient evidence from experimental animal models but limited human, epidemiological and mechanistic evidence (5) (6). IARC also concluded that there were too few studies and inconsistent results to comment on the evidence of sex steroid hormone alterations, especially among men (6).

Experimental evidence shows that circadian disruption due to night shift work is associated with a wide range of diseases, including cancer, cardiovascular diseases, and metabolic disorders (7, 8). Multiple epidemiological studies have examined changes in melatonin among night shift workers (9), however fewer studies have focused on sex hormone-related changes in this population (1, 10, 11). Sex steroids have wide-ranging impacts, including influencing cancer development and progression (12). Prostate cancer, the most common cancer among men, is hormone-dependent (13), and there is mounting evidence of an association between night shift work and this cancer (14–16). A possible mechanism to explain the elevated prevalence of hormone-related cancers among night shift workers may be through the light-induced suppression of melatonin and alterations in sex steroid hormone rhythms (17–20).

Studies that have examined melatonin and sex steroid hormone changes among night shift workers have included populations with permanent night shift work (1) or fast rotating night shift work (10, 11) compared to permanent day shift workers, with findings suggesting that sex-hormone rhythms, as well as melatonin rhythms, varied with different shift schedules and light conditions (1, 3). Furthermore, most previous studies have compared hormonal production between subjects rather than focusing on individual changes within each participant (1, 3, 10, 11).

The HORMONIT study aimed to evaluate hormone-related changes in melatonin and sex steroids in a population of male rotating shift workers working both day and night shifts in a slow backward rotation. We hypothesized that night shift work would be associated with changes in the timing and total production of melatonin and sex steroid hormones.

## Methods

### Study population

This study included male rotating shift workers from a car factory in Barcelona, Spain. Workers rotated backwards (counterclockwise) through shifts: night shift (22:00–06:00 hrs), evening shift (14:00–22:00 hrs), and early morning shift (06:00–14:00 hrs). The majority of workers were assembling/mounting car parts, while others were supervisors, painters or polishers and drivers. Workers worked five-day work weeks (Monday–Friday) with two days off (Saturday and Sunday). Workers completed a given shift in 3 week stretches before switching to a different shift. After the 3-week rotation, they began the next rotation with no extra time off besides the standard weekend. We collected data from participants twice. The first time point of sample collection occurred when participants were working an early morning shift near the end of their 3-week early morning shift rotation (2^nd^ or 3^rd^ week). The second point of sample collection occurred when participants were working a night shift near the end of a 3-week night shift rotation (2^nd^ or 3^rd^ week). We did not collect samples from participants on Mondays to avoid some potential shifting of circadian rhythms due to nighttime sleeping during the weekend days off.

To be eligible for the study, participants had to be 18–65 years of age. We excluded participants with a prior history of cancer. The Parc de Salut Mar Clinical Research Ethics committee approved the study (#2015/6351). Participants received a leaflet with study information, and all participants signed an informed consent form. A total of 71 men volunteered for this study. After checking eligibility criteria, 7 participants were found to be ineligible and 8 withdrew prior to study start due to the time commitment involved. Ultimately, 56 participants were enrolled in the study, of whom 51 completed all parts of data collection. From the 51 participants, 7 lacked complete melatonin and sex steroid hormone data at both time points, resulting in 44 participants who had complete data and were included in the present analyses (supplementary material, www.sjweh.fi/article/3991, figure S1).

### Exposures

At the start of the early morning shift, participants were interviewed and data on demographics, work-related information, smoking, alcohol, caffeine, dietary habits, medical history and medication use, and sleep-related information were collected.

### Light exposure assessment

In addition to questionnaire-collected data, each participant was given a light sensor (HOBOware, Onset Computer Corporation). Similar to a previous study by Papantoniou et al (3), participants wore a HOBOware light intensity data logger that continuously recorded their ambient light exposure every 12–15 seconds over an approximate 24-hour period corresponding to the days when participants collected urine. The logger was relatively small in size (5.8 × 3.3 × 2.3 cm) and light in weight (18 g) and was worn at shoulder level in order to obtain measurements of light that would approximate the amount of light reaching the retina. During sleep, participants were instructed to place the logger on a bedside table with the sensor facing upwards and while showering, participants were instructed to leave the logger nearby in the bathroom again with the sensor facing upwards. The loggers recorded relative light intensity within the range of 0–320 000 lux and were designed for indoor and outdoor settings. Overall, the mean and median 24-hour light exposure as well as mean and median light exposure during working hours were estimated.

### Outcomes

Participants provided urine samples from all natural urine voids over an approximate 24-hour period on two separate working days (one during the night shift and one during the early morning shift). Participants collected urine samples in 50 mL plastic tubes, labeled with the time and date of each collection. We advised participants to keep the urine samples in a refrigerator immediately after collection and to send them to the receiving laboratory by courier mail 1 or 2 days later. The urine aliquots for aMT6s analyses were stored at -80°C and the aliquots for steroid analyses were stored at -20°C until analyses. A total of 682 urine samples were collected and analyzed. During the early morning shift, participants collected a median of 7 (interquartile interval 6-8) urine samples, and during the night shift a median of 7 (interquartile interval 6-9) samples were collected. We required participants to have ≥ 4 urine samples during each 24-hour day. This resulted in the exclusion of early morning shift hormone data for 3 participants and night shift hormone data for 2 participants.

### Determination of the levels of sex steroid hormones, their main metabolites, and enzymes

Urinary concentrations of sex steroid hormones (androgens, estrogens and progestogens) and their main metabolites were determined by liquid chromatography tandem mass spectrometric (LC-MS/MS) methods previously developed in our group (21–23). The activity of several related enzymes was estimated using the ratio between the metabolite and the hormone concentrations (supplementary table S1). A global estimation of the metabolism was also estimated by dividing the levels of each hormone by the sum of all its corresponding metabolite levels. Specifically, we examined androgen hormones, metabolites and related enzymes (12 analyzed), progestogen hormones and metabolites (5 analyzed), and estrogen hormones, metabolites and related enzymes (4 analyzed).

Urinary concentrations of sex steroid metabolites (excreted as both unconjugated and glucuronoconjugated) were determined based on a previously validated method (22). Briefly, urine (0.5 mL) was mixed with 50 µL of the internal standard solution, 0.5 mL of sodium phosphate buffer (1M, pH 7) and 30 µL of β-glucuronidase from E. coli. After hydrolysis (1 hour at 55 °C), 2 mL of saturated sodium chloride solution, 250 µL of 25% of potassium carbonate and 6 mL of ethyl acetate were added and a liquid-liquid extraction was performed. The organic layer was extracted and evaporated to dryness (40 °C, <15 psi). Dried extracts were reconstituted with 150 μL of water: methanol (9:1) and 10 μL were injected into the LC-MS/MS system. The limit of detection of the method was in the range 0.2–10 ng/mL and the coefficient of variation (CV) at three concentration levels were in the range 80–120%. Intra- and inter-day precisions typically <20% were obtained for the detection of urinary steroids (22).

Urinary steroid cysteinyl metabolites were determined as previously reported (23). Briefly, 0.5 mL of urine was mixed with 50 μL of the internal standard (methandienone, 1 μg/mL), and basified by addition of 300 μL of potassium hydroxide (6 M). The mixture was heated at 60 °C for 15 minutes, followed by a liquid–liquid extraction with 6 mL of *tert*-butyl methyl ether. The organic layer was separated and evaporated. The residue was dissolved into 150 μL of a mixture of water:acetonitrile (1:1, v/v) and 10 μL were injected into the LC–MS/MS system. The limit of detection of the method was in the range 0.001–0.05 ng/mL and the CV% at three concentration levels were in the range 85–115%. Intra- and inter-day precisions typically <20% were obtained for the detection of urinary steroid cysteinyl metabolites (23).

### Determination of the levels of aMT6s

We also measured urinary 6-sulfatoxymelatonin (aMT6s) concentrations, the major melatonin metabolite by radioimmunoassay (Stockgrand Ltd, University of Surrey, UK) (24). Urine samples were analyzed in duplicate and all samples from the same participant were included in the same assay. The inter-assay coefficients of variation were 6.9% at 2.4 [± standard deviation (SD)] 0.2 ng/mL, 7.6% at 11.3 ±SD 0.9 ng/mL, and 6.9% at 21.1 ±SD 1.4 ng/mL. Limit of detection was 0.33 ng/ml.

### Determination of the levels of creatinine

Creatinine levels were determined in all urine samples by the same laboratory (Stockgrand Ltd, University of Surrey, UK). Levels were determined automatically using an IL600 analyzer (Randox Laboratories Ltd, UK). The limit of detection of the assay was 1.5 mmol/L and interassay variability was 3.2% at 6.95 ±SD 0.22 mmol/L and 3.8% at 14.01 ±SD 3.78 mmol/L. All aMT6s values and sex steroid metabolites were creatinine standardized and reported as ng metabolite/mg creatinine.

### Covariates

We examined within-person variations in hormone levels for the early morning shift period versus the night shift period. Because of this within-person comparison approach, adjustment was not required for many covariates traditionally adjusted for in shift work circadian rhythm studies. However, variation in daylight hours is known to impact circadian rhythms (25, 26). Because urine samples were taken at two time points, that in some cases differed by several weeks (median 45 (±SD 43) days), we adjusted analyses for length of daylight (using values available from the National Oceanic and Atmospheric Association calculator and inputting the latitude 41^o^ 230 N and longitude 2^o^ 100 E for Barcelona) (27).

To define chronotype in our study, the aligning of an individual with greater morningness or eveningness tendencies (28), participants responded to the Munich Chronotype Questionnaire for shift workers (MCTQShift) (29) both at the beginning of an early morning shift and at the beginning of a night shift. Chronotype (MSF_corr_) was estimated as the mid-sleep time on free days (MSF = [sleep onset on free day + sleep duration on free day]/2), corrected for oversleep on free days compared to working days (MSF_corr_ = MSF – [sleep duration on free day-sleep duration on a working day]/2). Sleep duration was calculated from patient reported sleep onset and offset. We assessed chronotype as a categorical variable where categories were built using tertiles of the distribution in our population [morning type: MSF <04:00 hr, neither type: MSF (04:00–04:50) hr, evening type: MSF >04:50 hr] (30).

### Statistical analysis

We compared within-participant hormone levels during the night shift period to hormone levels collected during the early morning shift period as the reference.

To evaluate the rhythm of all hormones and their metabolites, we applied individual cosinor analysis, a procedure for fitting a sinusoidal curve. This method extrapolates values of hormones collected throughout the 24-hour day to plot the full diurnal rhythm of a given hormone or metabolite for participants (31). For each participant, we derived the acrophase (peak time), mesor (24-hour mean), amplitude (doubling the amplitude is a measure of the extent of predictable change within a cycle) and the area under the curve (AUC, total production) of the metabolites (32). To check the cosinor-derived parameters, we examined the percentage of variability accounted for by the cosine curve (100% indicates that all data points fall on the cosine curve, supplementary tables S2 and S3). 6-Sulphatoxymelatonin and the majority of the steroid hormones and metabolites had moderate or higher fits. However, in some cases for a given participant and shift, the R-squared values indicated low fits. We described the 24-hour total production [geometric mean (GM) and SD] and peak time [GM and 95% confidence interval (CI)] for all outcomes in participants both during their early morning shift and during their night shift.

Using generalized linear models, we examined associations between shift work schedule and log-transformed 24-hr peak time (acrophase) and total production. We applied log transformation to achieve a normal distribution of the variables. Then, we applied linear mixed models to evaluate the differences in the acrophase (presented as geometric mean differences [GMD]) and the AUC (presented as the geometric mean ratio [GMR]) of hormones and their metabolites comparing the values from the night shift to those from the early morning shift.

Additionally, we examined if there was evidence of effect modification for the aMT6s results based on chronotype (morning type, evening type, neither type), categories of self-reported cumulative duration (years) of night shift work history, or based on level of light exposure (low, moderate, high) during work hours of night shift work from light sensor data. For cumulative duration of night shift work history, we created two categories based on the population mean exposure time (table 1). For level of light exposure during night shift work hours, we created these categories based on tertials of the population median exposure levels (low: median 37.0 (IQR 30.9–40.5), moderate: median 63.2 (IQR 53.7–72.6), high: median 114.6 (IQR 94.7–156.8). To examine if there was evidence of effect modification, we introduced an interaction term into the model and reported effect estimates across different chronotypes, categories of cumulative duration of night shift work history, and light exposure levels during night shifts.

**Table 1:** Characteristics of study population (N=44). [SD=standard deviation; IQR=interquartile range; BMI=body mass index.]

	Mean (SD)	Median (IQR)	%
Age (years)	38 (8)		
Height (cm)	174 (8)		
Weight (kg)	80 (13)		
BMI (kg/m^2^)			
<25			45
25–30			34
≥30			21
Education			
Primary			20
Professional			80
Smoking			
Never			41
Former			21
Current			36
Chronotype			
Morning			34
Neither			34
Evening			32
Self-reported sleep duration			
On a work day	6 (1)		
On a non-work day	8 (2)		
Light exposure during early morning shift (lux), median (IQR)		488 (235–957)	
Light exposure during night shift (lux), median (IQR)		78 (40–95)	
Cumulative duration of shift work history (years) ^a^	10 (6)		
Physical activity level of work			
Sedentary			4
Low intensity			16
Moderately active			50
Very active			31
Diagnosed with a chronic disease ^b^			25
Currently use medication ^c^			21
Number of days into shift at the time of collection, median (IQR)			
Early morning		17 (16–18)	
Night		16 (16–17)	
Number of consecutive days worked prior to collection, median (IQR)			
Early morning		2 (2–3)	
Night		2 (1–2)	
Average number of days between sampling time points		45 (43)	
Average minutes of daylight difference between early morning and night shift		72 (67)	

a Cumulative duration of shift work history missing for 15 (30%) participants.

b Reported chronic conditions include: anemia, asthma or allergy, cholesterol, hepatitis C, hypertension, attention deficit disorder, uric acid, anxiety.

c Medications asked about include: use of aspirin, hypnotics, melatonin or a sedative.

Finally, because some participants were sampled during the 2^nd^ week and others in the 3^rd^ week and because the day of sample collection (Tuesday–Friday) during a given week differed between participants, we conducted some sensitivity analyses. The week of the shift (2^nd^ or 3^rd^) would mean that participants would potentially be more or less adapted to their shift schedule, and the distance from the weekend (Tuesday-Friday) may similarly influence hormone levels based on for example possible sleep habit differences on a weekend versus a work day. To examine these factors, we reported estimates from a) an analysis that adjusted for the day of the shift the participant was in at the time of sampling (1–21 days with the first Monday of that shift considered day 1) and b) an analysis that adjusted for the number of prior consecutive days of that shift (ie, if sampled on a Wednesday, the number of prior consecutive days of the shift would be 2 since the weekend).

## Results

The mean age of the population was 38 (SD 8) years, and participants had worked night shifts for 10 (SD 6) years. More than half of the population had a BMI ≥25 kg/m^2^ (55%) and were former or current smokers (57%). The average light exposure during work on an early morning shift was 913 (SD 1396) lux while during a night shift the corresponding value was 78 (SD 58) lux. Sample collections were done on an early morning shift among 37 participants (84%) while 7 (16%) participants’ samples were collected on a night shift. Samples were collected for participants at a median of 17 days into the 21-early morning shift rotation (IQR 16–18) for the day shift and on the 16^th^ (IQR 16–17) day of the night shift. Furthermore, participants had worked a median of 2 (IQR 2–3) consecutive days prior to when the early morning shift samples were collected and a median of 2 (IQR 1–2) consecutive days prior to when the night shift samples were collected. The average time between when participants collected data during the early morning shift period and when they collected data during the night shift period was 45 (SD 43) days (table 1).

**Figure 1:**
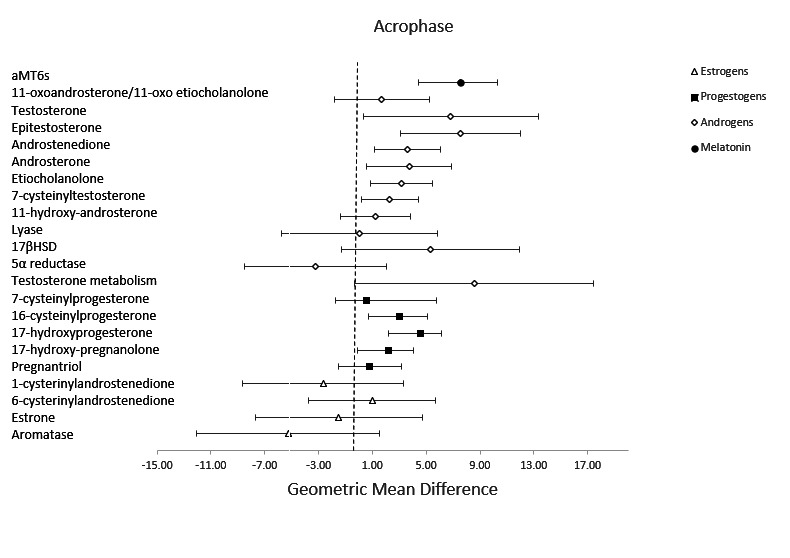
This figure presents the estimated hours of difference (geometric mean difference [GMD] and 95% confidence interval) in individual hormones peak time (acrophase) between the early morning shift and night shift, adjusting for length of daylight. The differences are viewable for melatonin and each group of sex steroid hormones including androgens, progestogens and estrogens.

**Figure 2:**
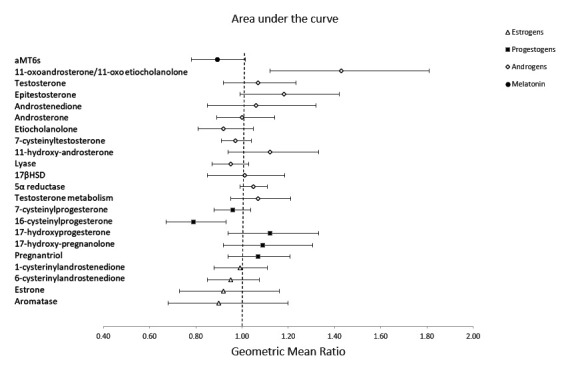
This figure presents the estimated geometric mean ratio (GMR) and 95% confidence intervals for the individual total hormone production (AUC) during the early morning shift and night shift, adjusted for length of daylight. The differences are viewable for melatonin and each group of sex steroid hormones including androgens, progestogens and estrogens.

In analyses adjusted for hours of daylight, we observed a later acrophase peak in aMT6s during the night shift compared to the early morning shift (supplementary figure S2). In addition, the acrophase shifted for aMT6s during the night shift compared to the early morning shift (GMD 7.53, 95% CI 4.46–10.60) (table 2, figure 1). We also examined changes in overall hormone and metabolite levels, using the AUC values. The total production of aMT6s was lower in samples collected during the night shift (GMR 0.89, 95% CI 0.78–1.01) compared to during the early morning shift, but this difference did not reach statistical significance (table 3, figure 2).

**Table 2:** Peak time (acrophase) of hormone and metabolite production [geometric mean (GM) and 95% confidence interval (CI)] during early morning and night shift and estimated hours of difference in hormone and metabolite’s peak production time [GMD=geometric mean difference (GMD) and 95% CI] between night and early morning workers shifts.

	Peak time (acrophase), hours:minutes ^a^				Estimated hours difference in peak time ^b^	
	
	GM early morning	95% CI	GM night	95% CI	GMD	95% CI
aMT6s	4:38	4:14–5:05	13:37	12:49–14:29	7.53	4.46–10.60
Androgens						
11-oxoandrosterone/11-oxoetiocholanolone	9:57	7:36–13:01	11:42	9.24–14:33	1.70	-1.81–5.21
Testosterone	5:35	3:48–8:11	13:38	11:10–16:39	6.83	0.34–13.32
Epitestosterone	6:05	4:43–7:52	15:33	14:09–17:05	7.54	3.09–12.00
Androstenedione	8:40	7:01–10:41	13:35	11:50–15:37	3.60	1.13–6.08
Androsterone	9:18	7:18–11:52	13:51	11:48–16.14	3.72	0.55–6.89
Etiocholanolone	9:11	7:41–10:58	13:21	11:28–15:32	3.18	0.88–5.48
7-cysteinyltestosterone	12:11	10:55–13:36	14:55	12:31–17:46	2.30	0.18–4.42
11-hydroxyandrosterone	10:37	8:51–12:43	11:39	8:47–15:27	1.23	-1.39–3.86
Lyasec	9:42	7:41–12:14	10:34	7:26–15:02	0.07	-5.73–5.87
17β-hydroxysteroid dehydrogenase (17βHSD) ^c^	4:22	2:43–7:00	9:44	7:14–13:05	5.33	-1.28–11.92
5α-reductase ^c^	10:31	8:05–13:40	7:43	5:20–11:11	-3.22	-8.51–2.06
Testosterone metabolism ^c^	3:52	2:40–5:37	10:44	7:56–14:30	8.56	-0.33–17.44
Progestogens						
7-cysteinylprogesterone	10:15	8:23–12:32	9:55	7:14–13:36	0.55	-4.66–5.75
16-cysteinylprogesterone	7:40	6:30–9:02	13:08	10:25–16:34	3.03	0.93–5.13
17-hydroxyprogesterone	8:43	8:00–9:29	14:37	13:04–16:22	4.54	2.92–6.16
17-hydroxy-pregnanolone	7:31	5.36–10.23	14:17	10:59–18:35	2.19	0.33–4.05
Pregnantriol	9:50	7:41–12:14	9:55	7:02–13:58	0.82	-1.49–3.13
Estrogens						
1-cysteinylandrostenedione	12:06	11:18–12:57	9:54	6:21–15:26	-2.66	-8.66–3.34
7-cysteinylandrostenedione	9:44	7:59–11:52	10:08	7:01–14:37	0.99	-3.74–5,71
Estrone	9:55	6:50–14:23	9:05	6:34–12:34	-1.50	-7.70–4.69
Aromatase ^c^	11:46	9:04–15:15	5:47	3:50–8:46	-5.26	-12.04–1.53

a Peak time is expressed in local time.

b Results adjusted for hours of daylight.

c Enzymes activities were estimates as shown in supplementary table 1.

**Table 3:** Estimated geometric mean (GM) and standard deviation (SD) for total production (AUC) during early morning shift and night shift and estimated geometric mean ratio (GMR) and 95% CI of hormone and metabolite production between night and early morning workers shifts.

	Unadjusted				Adjusted ^a^	
	
	GM early morning	SD	GM night	SD	GMR	95% CI
aMT6s	168.9	0.52	151.3	0.56	0.89	0.78–1.01
Androgens						
11-oxoandrosterone/11-oxoetiocholanolone	4655.6	0.72	6033.0	0.97	1.43	1.12–1.81
Testosterone	427.1	0.92	451.0	0.82	1.07	0.92–1.23
Epitestosterone	533.5	0.73	609.6	0.65	1.18	0.99–1.42
Androstenedione	28.7	0.71	29.8	0.89	1.06	0.85–1.32
Androsterone	29 320.1	0.53	28 050.7	0.55	1.00	0.89–1.14
Etiocholanolone	22 640.8	0.55	20 797.6	0.58	0.92	0.81–1.05
7-cysteinyltestosterone	34.6	0.59	32.8	0.57	0.97	0.91–1.04
11-hydroxyandrosterone	8845.6	0.55	9102.5	0.63	1.12	0.94–1.33
Lyase ^b^	92.0	0.41	86.2	0.47	0.95	0.87–1.03
17β-hydroxysteroid dehydrogenase (17βHSD) ^b^	369.9	0.98	373.8	1.16	1.01	0.85–1.19
5α-reductase ^b^	31.7	0.46	32.6	0.45	1.05	0.99–1.11
Testosterone metabolism ^b^	0.2	0.89	0.2	0.92	1.07	0.95–1.21
Progestogens						
7-cysteinylprogesterone	5.7	0.87	5.4	0.89	0.96	0.88–1.04
16-cysteinylprogesterone	37.8	1.41	31.7	1.44	0.79	0.67–0.93
17-hydroxy Progesterone	8.1	0.45	8.7	0.54	1.12	0.94–1.33
17-hydroxypregnanolone	1705.3	0.67	1749.9	0.68	1.09	0.92–1.30
Pregnantriol	8951.8	0.62	9161.1	0.64	1.07	0.94–1.21
Estrogens						
1-cysteinylandrostenedione	2.5	0.52	2.4	0.51	0.99	0.88–1.11
7-cysteinylandrostenedione	257.2	1.05	235.2	1.02	0.95	0.85–1.07
Estrone	9.7	1.48	7.8	1.47	0.92	0.73–1.16
Aromatase ^b^	8.7	1.54	7.1	1.60	0.90	0.68–1.20

a Results adjusted for hours of daylight.

b Enzymes activities were estimates as shown in supplementary table S1.

In addition to changes in aMT6s, we observed a later acrophase peak in sex steroid hormones and their metabolites during the night shift compared to the early morning shift (supplementary figure 2). The acrophase was shifted for several androgens: testosterone (GMD 6.83, 95% CI 0.34–13.32), epitestosterone (GMD 7.54, 95% CI 3.09–12.00), androstenedione (3.60, 95% CI 1.13–6.08), androsterone (GMD 3.72, 95% CI 0.55–6.89), etiocholanolone (GMD 3.18, 95% CI 0.88–5.48) and 7-cysteinyltestosterone (2.30, 95% CI 0.18–4.42), and several progestogens: 16-cysteinylprogesterone (GMD 3.03, 95% CI 0.93–5.13), 17-hydroxyprogesterone (4.54, 95% CI 2.92–6.16), and 17-hydroxypregnanolone (GMD 2.19, 95% CI 0.33–4.05) (table 2, figure 1). There was no association between night shift work and the acrophase of estrogens. When examining the total production of sex steroid hormones, we observed a higher production of 11-oxoandrosterone/11-oxoetiocholanolone (GMR 1.43, 95% CI 1.12–1.81), and a lower production of 16-cysteinylprogesterone (GMR 0.79, 95% CI 0.67–0.93) during the night shift compared to their early morning shift levels (table 3, figure 2).

In our analyses examining possible effect modification in aMT6s levels during the night shift compared to the early morning shift by chronotype, cumulative night shift work history and light during night work, our results suggested that those with greater levels of light exposure during night shifts experienced a larger shift in acrophase during the night shift (GMD 7.32, 95% CI 4.18–10.47 for low light, GMD 8.60, 95% CI 4.68–12.52 for moderate light and GMD 10.70, 95% CI 5.67–15.64 for high light). We also found that those with greater levels of light exposure during the night shifts experienced lower levels of aMT6s production compared to the early morning shift (GMR for high light exposure 0.73, 95% CI 0.60–0.88 compared to GMR 0.89, 95% CI 0.73–1.07 and GMR 1.01, 95% CI 0.83–1.22 for moderate and high light, respectively) while those with a shorter duration of night shift work history did not have a difference in total production of aMT6s (supplementary table S4). There was no evidence of differences in aMT6s production during the night compared to the early morning shift based on chronotype or cumulative duration of prior night shift work.

Finally, results from the two sensitivity analyses with additional adjustment for the day number of the shift and the number of prior consecutive days in the shift produced results similar to the primary analyses (supplementary table S5 and S6).

## Discussion

In this study of slow, backward rotating shift workers, the acrophase time of aMT6s, androgens and progestogens were significantly delayed during the night shift compared to the early morning shift. Alterations in the total production of several hormones were also apparent.

We found that the acrophase of aMT6s was delayed by several hours (4.5 hours) during the night shift work period. The phase shift of aMT6s between the early morning and night was found to be larger among participants who had greater levels of light exposure during night shift work, which is in agreement with previous studies (3, 33, 34). In an earlier study, the acrophase of melatonin was shifted by 3 hours (3). Furthermore, in the present study, overall aMT6s production was slightly lower during night shifts than early morning shifts, however the effect estimate was not pronounced. In the earlier study (3), aMT6s production was decreased among night shift workers with a mean production that was 33% lower among night shift workers compared to day shift workers. In the present study, total aMT6s production levels were somewhat lower (10%), but CI overlapped one (3). Several aspects should be considered when comparing results from the present study to other melatonin biomarker studies in night shift workers. Firstly, the population in the present study worked in slow rotating shifts, with samples collected during the second or third week of a 3-week rotation. This was done because we were interested in examining how hormone levels and timing were altered after adaptation to the shift schedule sets in to some degree. Previous research suggests that extended periods of night shift work are needed to achieve adaptation (35, 36), though many individuals do not appear to be capable of complete adaptation (37). Slow rotating shifts are very different from fast rotating shifts, such as those included in our earlier research (1, 3) and are expected to influence hormone rhythms to a different degree because of adaptation to the shift schedule. An additional consideration should be given to the timing of the shifts. It is possible that because our reference group was an early morning shift (beginning at 06:00 hrs) while the other analysis included a less extreme day shift comparison, that we may observe a larger acrophase delay because the contrast between night and early morning is bigger than the one between a day and night shift. However, in any case, the change in aMT6s between night and early morning workers is important because melatonin is a major driver of circadian rhythms, and it also has anti-cancer properties (38, 39). Because of this, alterations in melatonin production among night shift workers is a suggested key pathway explaining the carcinogenicity of shift work.

In the present study, the acrophase of many androgen hormones, metabolites and androgen-related enzymes was shifted during the night shift compared to the early morning shift. There is limited prior epidemiological literature focusing on the circadian rhythm of androgens among male night shift workers. Androgens are widely implicated in cancer progression, with research showing that androgen deprivation is accompanied by tumor regression (40). Besides the phase shift for many of the steroids examined, there was over production of the main metabolites of 11-oxygenated androgens (11-oxoandrosterone/11-oxoetiocholanolone) during the night shift. These adrenal-derived androgens have emerged as major components of several disorders (41–43). Among these disorders, 11-oxygenated androgens are the predominant circulating androgens in castration resistant prostate cancer (CRPC) (44), and they are the preferred substrate for AKR1C3 (an enzyme that catalyzes the reduction of weak androgens to more potent androgens), which is increased in CRPC tumors (45). Thus, 11-oxygenated androgens have been identified as key components in the development and progression of castration resistant prostate cancer (46, 47). Our results pointed to an over production of these androgens during the night-shift, suggesting a potential mechanism between night-shift and prostate cancer development and progression.

We also found changes in the acrophase of several progestogens and a lower production of the adrenal progestogen 16-cysteinylprogesterone warranting further study of progestogens among male night shift workers. Several prior studies have examined changes in these hormones among female night shift nurses, with results showing higher levels of estrogen and progesterone among women working rotating night shifts compared to day shift workers (11). While progesterone has been extensively studied in relation to female cancers, progesterone also has essential functions in male physiology, and the presence of progesterone receptors has been confirmed in several cancers, including prostate cancer (48). Furthermore, the co-expression of steroid hormone receptors in hormone-dependent cancers is widespread and these receptors may interact to a large degree, possibly impacting tumor growth and progression. In the present study, estrogen levels were unchanged during the night shift period.

Both 11-oxoandrosterone/11-oxoetiocholanolone, which was over produced during the night shift, and progestogen 16-cysteinylprogesterone, which was under produced during the night shift, are adrenal-derived steroids (21, 46), suggesting that the production of adrenal steroids is altered by night shift work. The adrenal gland plays a pivotal role in CRPC as evidenced by the relevance of adrenal-derived androgens (46, 47) and by suppression of tumor growth in animal models by surgical adrenalectomy (49). The essential role of the adrenal peripheral clock in harmonizing the rhythm of adrenal glucocorticosteroid production is well established (50, 51). However, the influence of this clock in the production of adrenal androgens/progestogens and its consequences in health and disease are as yet poorly understood and require further investigation.

There are some limitations of the present study including the small sample size, however, by having repeated measures for each participant this was mitigated to some extent. In addition, the reference samples came from workers engaged in early morning shift work. These participants began work early (at 06:00 hrs), and we expect hormone levels measured during these early morning shifts were also altered in part by this early work start time and do not fully represent the natural 24-hour rhythm that may be seen if our comparison group had included participants working more traditional hours who did not have to wake up so early for a morning shift. Additionally, we have extrapolated steroid production by measuring urinary metabolites. This strategy might underestimate alterations in metabolism as underlying cause of the observed alterations. Finally, the HOBO light logger has been found to underestimate light at low light levels and therefore the light levels during the night shift in secondary analyses may not be as low as reported. This study also has several strengths including the collection of all urine voids during 24 hours (approximately 7) and many parameters that are important for circadian research including light exposure, chronotype and information on prior shift work history. In addition, by comparing participants to themselves, we minimized confounding.

In conclusion, night shift work was associated with a delay in the peak time of aMT6s and several sex hormones as well as being associated with changes in total hormone production of androgens and progestogens. These findings provide further insight into mechanistic pathways that may explain the association of night shift work with prostate or other hormone-dependent cancers.

## Supplementary material

Supplementary material
