# Supplementary material for: Changes in melatonin and sex steroid hormone production among men as a result of rotating night shift work – the HORMONIT study
Source: Scand J Work Environ Health. 2021 Dec 30;48(1):41–51. doi: 10.5271/sjweh.3991 (PMC8729163; doi:10.5271/sjweh.3991)

Changes in melatonin and sex steroid hormone production among men as a result of rotating night shift work – the HORMONIT study[1]

by Barbara N Harding, PhD,[2] Gemma Castaño-Vinyals, PhD, Anna Palomar-Cros, MPH, Kyriaki Papantoniou, PhD, Ana Espinosa, MS, Debra J Skene, Pharm, MSc, PhD, Benita Middleton, PhD, Alex Gomez-Gomez, MS, José Maria Navarrete, Patricia Such Faro, Antonio Torrejón, Manolis Kogevinas, MD, PhD, Oscar J Pozo, PhD

1. *Supplementary tables and figures*
2. *Correspondence to: Barbara Harding, Barcelona Institute for Global Health - Campus MAR, Doctor Aiguader, 88, 08003 Barcelona, Spain. [E-mail: Barbara.harding@isglobal.org]*

Supplementary Table S1.

**Enzyme**	**Estimation**
Lyase	(Androsterone+Etiocholanolone)/(Pregnantriol + 17-hydroxypregnanolone)
17βHSD	Testosterone/Androstenedione
5α-reductase	Androsterone/Etiocholanolone
Testosterone metabolism	(Androsterone +Etiocholanolone+ Androstenedione)/Testosterone
Aromatase	Estrone/Androstenedione

Supplemental Table 2: Summarizing the percentage of variability accounted for by the cosine curve for aMT6s and androgens by participant and by shift										
Person	Shift	aMT6s	11-oxo-androsterone/11-oxo-Etiocholanolone	Testosterone	Epitestos terone	Androsten dione	Andro sterone	Etiochol anolone	6-cysteinyl-testosterone	11-hydroxy-androsterone
1	Early morning	0.86	0.82	0.67	0.85	0.42	0.78	0.83	0.47	0.38
1	Night	0.87	0.02	0.50	0.67	0.46	0.43	0.26	0.28	0.84
2	Early morning	0.91	0.42	0.21	0.02	0.07	0.03	0.04	0.29	0.12
2	Night	0.96	0.82	0.59	0.74	1.00	0.95	0.83	0.58	0.87
3	Night	0.55	0.72	0.77	0.76	0.78	0.79	0.75	0.64	0.40
3	Early morning	0.81	0.36	0.44	0.24	0.17	0.47	0.63	0.41	0.23
4	Night	0.93	0.97	0.99	0.72	0.69	0.93	0.97	0.81	0.99
4	Early morning	0.93	0.46	0.34	0.70	0.33	0.73	0.66	0.79	0.64
5	Night	0.89	0.76	0.62	0.80	0.99	0.97	0.75	0.37	0.37
5	Early morning	0.82	0.68	0.94	0.94	0.64	0.79	0.92	0.38	0.96
6	Night	0.97	0.51	0.93	0.93	0.78	0.08	0.87	0.63	0.61
6	Early morning	0.77	0.78	0.98	0.77	0.32	0.87	0.87	0.26	0.14
7	Early morning	0.61	0.47	0.53	0.55	0.45	0.47	0.44	0.39	0.52
7	Night	0.72	0.75	0.90	1.00	0.24	0.01	0.21	0.88	0.77
8	Night	0.70	0.75	0.27	0.71	0.62	0.33	0.98	0.82	0.56
8	Early morning	0.82	0.90	0.20	0.68	0.38	0.90	0.95	0.90	0.98
9	Night	0.73	0.38	0.50	0.58	0.61	0.40	0.41	0.80	0.45
9	Early morning	0.87	0.18	0.38	0.81	0.07	0.32	0.64	0.01	0.57
10	Night	0.99	0.76	0.82	0.84	0.88	0.45	0.24	0.85	0.64
10	Early morning	0.72	0.93	0.64	0.76	0.79	0.98	0.78	0.89	0.88
11	Night	0.80	0.15	0.81	0.95	0.88	0.79	0.80	0.32	0.52
11	Early morning	0.86	0.15	0.63	0.52	0.70	0.43	0.43	0.79	0.33
12	Night	0.81	0.09	0.67	0.75	0.40	0.06	0.09	0.16	0.24
12	Early morning	0.69	0.68	0.65	0.68	0.75	0.53	0.12	0.69	0.79
13	Night	0.91	0.09	0.06	0.03	0.04	0.17	0.07	0.52	0.18
13	Early morning	0.83	0.48	0.26	0.35	0.23	0.30	0.23	0.89	0.51
14	Night	0.76	0.39	0.47	0.77	0.57	0.44	0.47	0.25	0.72
14	Early morning	0.91	0.41	0.35	0.43	0.19	0.43	0.40	0.96	0.46
15	Night	0.86	0.02	0.49	0.37	0.54	0.29	0.47	0.07	0.81
15	Early morning	0.85	0.70	0.46	0.66	0.93	0.47	0.82	0.83	0.43
16	Night	0.92	0.59	0.54	0.35	0.30	0.26	0.39	0.40	0.42

16	Early morning	0.79	0.71	0.41	0.45	0.75	0.70	0.65	0.84	0.17
17	Night	0.81	0.66	0.28	0.64	0.57	0.54	0.69	0.13	0.79
17	Early morning	0.51	0.32	0.98	0.81	0.81	0.43	0.62	0.20	0.49
18	Night	0.80	0.41	0.83	0.96	0.79	0.86	0.83	0.82	0.32
18	Early morning	0.83	0.39	0.84	0.15	0.43	0.30	0.32	0.25	0.39
19	Night	0.93	0.73	0.96	0.91	0.89	0.90	0.75	0.71	0.81
19	Early morning	0.73	0.77	0.97	0.74	0.77	0.73	0.80	0.31	0.68
20	Night	0.91	0.46	0.61	0.68	0.57	0.77	0.30	0.59	0.66
20	Early morning	0.82	0.77	0.71	0.63	0.69	0.13	0.10	0.50	0.05
21	Night	0.72	0.84	0.64	0.89	0.62	0.15	0.30	0.08	0.65
21	Early morning	0.74	0.46	0.74	0.88	0.12	0.50	0.17	0.27	0.68
22	Night	0.88	0.25	0.71	0.65	0.41	0.62	0.36	0.91	0.81
22	Early morning	0.86	0.23	0.58	0.71	0.77	0.57	0.43	0.60	0.48
23	Night	0.88	0.60	0.70	0.57	0.19	0.37	0.44	0.81	0.71
23	Early morning	0.89	0.62	0.68	0.74	0.45	0.41	0.62	0.88	0.47
24	Night	0.31	0.34	0.13	0.32	0.51	0.68	0.62	0.77	0.70
24	Early morning	0.68	0.36	0.84	0.85	0.73	0.42	0.74	0.90	0.52
25	Early morning	0.95	0.55	0.39	0.60	0.21	0.50	0.54	0.92	0.50
25	Night	0.80	0.23	0.08	0.28	0.58	0.12	0.03	0.27	0.12
26	Early morning	0.88	0.11	0.41	0.82	0.10	0.07	0.48	0.20	0.79
26	Night	0.81	0.90	1.00	0.99	1.00	0.93	0.99	0.43	0.80
27	Early morning	0.97	0.60	0.76	0.92	0.87	0.45	0.57	0.42	0.22
27	Night	0.89	0.24	0.79	0.63	0.35	0.61	0.70	0.55	0.13
28	Early morning	0.93	0.06	0.09	0.88	0.87	0.93	0.31	0.54	0.93
28	Night	0.71	0.27	0.61	0.72	0.30	0.81	0.84	0.72	0.79
29	Early morning	0.91	0.33	0.69	0.69	0.47	0.44	0.69	0.77	0.44
29	Night	0.88	0.36	0.71	0.37	0.56	0.80	0.80	0.99	0.93
30	Early morning	0.82	0.27	0.39	0.97	0.35	0.44	0.61	0.70	0.31
30	Night	0.86	0.52	0.27	0.41	0.86	0.67	0.61	0.84	0.94
31	Early morning	0.90	0.32	0.28	0.38	0.56	0.79	0.49	0.67	0.55
31	Night	0.67	0.06	0.75	0.86	0.01	0.44	0.46	0.45	0.02
32	Early morning	1.00	0.98	0.97	0.74	0.97	0.91	0.65	0.39	0.99
32	Night	1.00	0.25	0.94	0.94	0.92	0.82	0.88	0.47	0.22
33	Early morning	0.98	0.08	0.80	0.46	0.13	0.19	0.67	0.01	0.04
33	Night	0.89	0.78	0.95	0.56	0.95	0.89	0.77	0.81	0.95
34	Early morning	0.61	0.05	0.92	0.53	0.63	0.12	0.09	0.08	0.04

34	Night	0.87	0.01	0.63	0.98	0.74	0.77	0.59	0.77	0.76
35	Early morning	0.92	0.44	0.04	0.11	0.34	0.26	0.39	0.61	0.76
35	Night	0.99	0.39	0.83	0.52	0.71	0.74	0.80	0.27	0.55
36	Early morning	0.21	0.13	0.62	0.95	0.79	0.99	0.73	0.62	0.08
36	Night	0.85	0.37	0.19	0.35	0.37	0.37	0.68	0.12	0.56
37	Early morning	0.96	0.19	0.45	0.16	0.72	0.66	0.15	0.31	0.96
37	Night	0.89	1.00	0.88	0.92	0.98	0.12	0.55	0.57	0.73
38	Early morning	0.97	0.68	0.67	0.23	0.61	0.87	0.97	0.86	0.96
38	Night	0.73	0.84	0.37	0.53	0.47	0.94	0.58	0.27	0.81
39	Early morning	0.90	0.29	0.49	0.67	0.92	0.63	0.65	0.68	0.80
39	Night	0.71	0.45	0.47	0.45	0.27	0.47	0.60	0.38	0.33
40	Early morning	0.79	0.05	0.42	0.33	0.70	0.44	0.23	0.13	0.92
40	Night	0.78	0.55	0.90	0.43	0.76	0.60	0.87	0.85	0.75
41	Early morning	0.89	0.71	0.81	0.81	0.65	0.75	0.77	0.86	0.58
41	Night	0.83	0.44	0.86	0.85	0.74	0.94	0.76	0.78	0.51
42	Early morning	0.84	0.32	0.82	0.88	0.49	0.48	0.45	0.09	0.36
42	Night	0.71	0.06	0.91	0.92	0.60	0.68	0.97	0.44	0.04
43	Early morning	0.92	0.11	0.84	0.77	0.92	0.98	0.71	0.72	0.18
43	Night	0.86	0.23	0.39	0.29	0.36	0.24	0.18	0.10	0.25
44	Night	0.94	0.50	0.12	0.40	0.23	0.33	0.21	0.09	0.19
44	Early morning	0.76	0.74	0.99	0.94	0.88	0.76	0.99	0.84	0.73

Supplemental Table S3: Summarizing the percentage of variability accounted for by the cosine curve for progestogens and estrogens by participant and by shift

ID	Shift	7-cysteinyl-progesterone	16-cysteinyl progesterone	_17-hydroxy-progesterone	17-hydroxy-pregnanolone	Pregnantriol	1-cysteinyl-androstenedione	7-cysteinyl-androstendione	Estrone
1	Early morning	0.46	0.26	0.05	0.76	0.61	0.70	0.28	0.49
1	Night	0.95	0.97	0.34	0.65	0.39	0.83	0.98	0.40
2	Early morning	0.08	0.57	0.01	0.03	0.01	0.49	0.61	0.71
2	Night	0.00	0.87	0.93	0.99	0.02	0.70	0.30	0.59
3	Early morning	0.56	0.54	0.52	0.78	0.72	0.51	0.37	0.02
3	Night	0.39	0.50	0.38	0.62	0.57	0.66	0.67	0.11
4	Early morning	0.51	0.89	0.83	0.97	0.52	0.73	0.82	0.69
4	Night	0.97	0.23	0.37	0.37	0.78	0.60	0.24	0.32
5	Early morning	0.32	0.52	0.71	0.77	0.93	0.81	0.10	0.06
5	Night	0.17	0.50	0.87	0.95	0.96	0.88	0.49	0.79
6	Early morning	0.58	0.62	0.67	0.93	0.90	0.65	0.23	0.37
6	Night	0.31	0.29	0.41	0.89	0.67	0.07	0.35	0.39
7	Early morning	0.06	0.04	0.46	0.38	0.09	0.35	0.16	0.51
7	Night	0.83	0.86	0.52	0.64	0.21	0.62	0.76	0.05
8	Night	0.80	0.17	0.32	0.97	0.98	1.00	0.78	0.93
8	Early morning	0.24	0.14	0.07	0.86	1.00	0.36	0.20	0.17
9	Night	0.13	0.36	0.35	0.55	0.19	0.14	0.03	0.73
9	Early morning	0.29	0.21	0.79	0.83	0.48	0.21	0.23	0.03
10	Night	0.43	0.53	0.88	0.45	0.26	0.19	0.52	0.76
10	Early morning	0.02	0.61	0.70	0.81	0.13	0.12	0.13	1.00
11	Night	0.15	0.47	0.73	0.78	0.81	0.81	0.45	0.57
11	Early morning	0.09	0.56	0.51	0.85	0.26	0.83	0.79	0.19
12	Night	0.15	0.45	0.31	0.89	0.07	0.36	0.27	0.77
12	Early morning	0.22	0.28	0.64	0.19	0.07	0.41	0.31	0.60
13	Night	0.59	0.42	0.25	0.20	0.16	0.77	0.81	0.33
13	Early morning	0.18	0.74	0.62	0.48	0.38	0.73	0.96	0.75
14	Night	0.35	0.53	0.84	0.64	0.54	0.62	0.34	0.79
14	Early morning	0.37	0.11	0.54	0.45	0.34	0.18	0.11	0.30
15	Night	0.18	0.55	0.34	0.27	0.15	0.37	0.30	0.19
15	Early morning	0.18	0.02	0.53	0.62	0.01	0.58	0.38	0.08
16	Night	0.53	0.62	0.08	0.15	0.40	0.86	0.56	0.32
16	Early morning	0.88	0.33	0.41	0.41	0.23	0.74	0.80	0.49

17	Night	0.09	0.19	0.88	0.48	0.50	0.48	0.08	0.31
17	Early morning	0.12	0.26	0.18	0.69	0.52	0.20	0.12	0.28
18	Night	1.00	0.91	0.93	0.97	0.82	0.96	0.96	0.73
18	Early morning	0.68	0.19	0.35	0.32	0.32	0.19	0.84	0.25
19	Night	0.21	0.54	0.56	0.85	0.76	0.82	0.83	0.09
19	Early morning	0.27	0.42	0.59	0.46	0.10	0.47	0.28	0.85
20	Night	0.25	0.10	0.53	0.66	0.37	0.38	0.14	0.67
20	Early morning	0.44	0.23	0.23	0.10	0.15	0.25	0.16	0.23
21	Night	0.03	0.84	0.78	0.81	0.72	0.86	0.29	0.55
21	Early morning	0.08	0.60	0.68	0.90	0.22	0.78	0.30	0.25
22	Night	0.93	0.88	0.60	0.82	0.80	0.97	0.85	0.11
22	Early morning	0.64	0.74	0.72	0.74	0.18	0.30	0.63	0.49
23	Night	0.73	0.46	0.41	0.55	0.55	0.84	0.85	0.69
23	Early morning	0.20	0.31	0.41	0.45	0.61	0.83	0.23	0.35
24	Night	0.59	0.27	0.32	0.38	0.73	0.55	0.45	0.34
24	Early morning	0.57	0.19	0.72	0.85	0.02	0.79	0.96	0.27
25	Night	0.66	0.23	0.45	0.62	0.61	0.40	0.41	0.37
25	Early morning	0.69	0.14	0.07	0.22	0.43	0.43	0.51	0.49
26	Night	0.94	0.96	0.57	0.00	0.06	0.97	0.96	0.79
26	Early morning	1.00	0.74	0.89	0.98	1.00	0.89	0.89	0.67
27	Night	0.26	0.38	0.64	0.70	0.27	0.55	0.34	0.42
27	Early morning	0.28	0.68	0.31	0.31	0.06	0.58	0.54	0.69
28	Night	0.31	0.80	0.82	0.95	0.56	0.73	0.42	0.75
28	Early morning	0.43	0.51	0.73	0.42	0.20	0.84	0.85	0.55
29	Night	0.61	0.20	0.35	0.43	0.88	0.40	0.12	0.29
29	Early morning	0.71	0.81	0.73	0.87	0.62	0.61	0.97	0.24
30	Night	0.41	0.07	0.52	0.98	0.84	0.92	0.71	0.73
30	Early morning	0.25	0.45	0.80	0.62	0.86	0.72	0.72	0.02
31	Night	0.81	0.10	0.24	0.53	0.50	0.00	0.64	0.70
31	Early morning	0.01	0.30	0.32	0.60	0.46	0.12	0.06	0.66
32	Night	0.45	0.59	0.76	0.37	0.98	1.00	0.42	0.47
32	Early morning	0.53	0.91	0.27	0.56	0.58	0.91	0.68	0.63
33	Night	0.56	0.46	0.19	0.20	0.53	0.06	0.04	0.59
33	Early morning	0.24	0.02	0.41	0.43	0.27	0.13	0.19	0.21
34	Night	0.15	0.35	0.33	0.22	0.02	0.76	0.53	0.94

34	Early morning	0.72	0.94	0.68	0.90	0.77	0.83	0.85	0.37
35	Night	0.53	0.23	0.30	0.01	0.38	0.61	0.56	0.66
35	Early morning	0.99	0.53	0.60	0.20	0.74	0.15	0.21	0.35
36	Night	0.58	0.61	0.09	0.77	0.77	0.86	0.82	0.60
36	Early morning	0.08	0.02	0.39	0.31	0.05	0.21	0.20	0.25
37	Night	0.12	0.33	0.71	0.13	0.18	0.57	0.32	0.74
37	Early morning	0.81	0.91	0.95	1.00	0.65	0.96	0.96	0.96
38	Night	0.83	0.41	0.81	0.47	0.76	0.57	0.78	0.66
38	Early morning	0.62	0.72	0.91	0.80	0.58	0.10	0.44	0.41
39	Night	0.77	0.30	0.41	0.82	0.36	0.86	0.73	0.04
39	Early morning	0.50	0.88	0.03	0.57	0.62	0.22	0.24	0.83
40	Night	0.23	0.27	0.91	0.20	0.01	0.08	0.42	0.02
40	Early morning	0.85	0.24	0.74	0.41	0.28	1.00	0.86	0.47
41	Night	0.19	0.69	0.32	0.79	0.83	0.86	0.82	0.33
41	Early morning	0.62	0.79	0.31	0.83	0.81	0.08	0.79	0.78
42	Night	0.33	0.40	0.58	0.59	0.64	0.50	0.02	0.35
42	Early morning	0.06	0.52	0.28	0.48	0.10	0.54	0.72	0.53
43	Night	0.79	0.91	0.38	0.12	0.23	0.94	0.66	0.13
43	Early morning	0.05	0.47	0.30	0.38	0.31	0.29	0.16	0.17
44	Night	0.06	0.25	0.49	0.02	0.09	0.28	0.14	0.88
44	Early morning	0.33	0.27	0.67	0.55	0.40	0.83	0.41	0.93

Supplemental Table S4: Effect modification of aMT6s peak levels (acrophase, presented as geometric mean differences [GMD]) and total production (AUC, presented as geometric mean ratios [GMR]) by chronotype/diurnal preference, cumulative history of night shift work and mean light levels during night shifts

	Adjusted results[a]			
	Acrophase GMD (95% CI)		AUC GMR (95% CI)	
aMT6s (all night vs early morning samples)	7.53	4.46-10.60	0.89	0.78-1.01
Diurnal preference[b]				
Morning (n=15)	7.67	4.37-10.97	0.93	0.76-1.13
Neither (n=15)	7.49	3.61-11.37	0.85	0.68-1.05
Evening (n=14)	7.42	3.77-11.08	0.88	0.71-1.09
Cumulative duration of night shift work (years)[c]				
<10 (n=17)	7.48	4.03-10.93	0.91	0.72-1.14
≥10 (n=14)	7.49	3.84-11.14	0.97	0.70-1.34
Light during night shift[d]				
Low (n=15)	7.32	4.18-10.47	1.01	0.83-1.22
Moderate (n=14)	8.60	4.68-12.52	0.89	0.73-1.07
High (n=14)	10.70	5.76-15.64	0.73	0.60-0.88

[a]Models adjusted for length of daylight

[b]Chronotype/diurnal preference is categorized by tertile of the MSF_{corr} score from the MCTQShift questionnaire(29)

[c]Duration of shift work data missing for 13 participants

[d]Mean light levels measured during work hours of the night shift using the HOBO light sensor, 1 participant with missing data

Supplemental Table S5: Area under the curve results from models adding additional adjustment for a) days of shift and b) consecutive days prior compared to results from the primary mixed models

AUC results									
	primary analysis adjusted			additional adjustment for days of shift			additional adjustment for prior consecutive days		
	GMR	95% CI upper	95% CI lower	GMR	95% CI upper	95% CI lower	GMR	95% CI upper	95% CI lower
**aMT6s**	0.89	0.78	1.01	0.88	0.77	1.01	0.83	0.73	0.96
**Androgens**									
11-oxoandrosterone/11-oxoetiocholanolone	1.43	1.12	1.81	1.48	1.15	1.89	1.45	1.11	1.88
Testosterone	1.07	0.92	1.23	1.05	0.90	1.22	1.11	0.94	1.30
Epitestosterone	1.18	0.99	1.42	1.15	0.95	1.39	1.19	0.98	1.45
Androstenedione	1.06	0.85	1.32	1.04	0.83	1.30	1.08	0.85	1.38
Androsterone	1.00	0.89	1.14	0.97	0.85	1.10	1.02	0.89	1.17
Etiocholanolone	0.92	0.81	1.05	0.91	0.79	1.05	0.95	0.82	1.11
7-cysteinyl-testosterone	0.97	0.91	1.04	0.97	0.91	1.05	1.01	0.93	1.09
11-hydroxy-androsterone	1.12	0.92	1.36	1.14	0.92	1.40	1.12	0.91	1.39
Lyase[c]	0.95	0.87	1.03	0.92	0.84	1.01	0.93	0.84	1.02
17β-hydroxysteroid dehydrogenase (17βHSD)[c]	1.01	0.85	1.19	1.02	0.85	1.22	1.05	0.87	1.27
5α-reductase[c]	1.05	0.99	1.11	1.02	0.97	1.08	1.03	0.96	1.09
Testosterone metabolism[c]	1.07	0.95	1.21	1.08	0.95	1.22	1.10	0.96	1.27
**Progestogens**									
7-cysteinylprogesterone	0.96	0.88	1.04	0.94	0.86	1.01	0.98	0.89	1.07
16-cysteinylprogesterone	0.79	0.67	0.93	0.79	0.66	0.93	0.83	0.69	1.00
17-hydroxyprogesterone	1.12	0.94	1.33	1.11	0.93	1.33	1.10	0.92	1.33
17-hydroxy-pregnanolone	1.09	0.92	1.30	1.07	0.90	1.28	1.12	0.93	1.35
Pregnantriol	1.07	0.94	1.21	1.06	0.93	1.21	1.09	0.95	1.25
**Estrogens**									
1-cysteinyl-androstenedione	0.99	0.88	1.11	0.96	0.85	1.09	1.02	0.89	1.16
7-cysteinyl-androstenedione	0.95	0.85	1.07	0.94	0.83	1.06	0.96	0.84	1.09
Estrone	0.92	0.73	1.16	0.93	0.73	1.19	0.97	0.75	1.26
Aromatase[c]	0.90	0.68	1.20	0.94	0.70	1.27	0.96	0.69	1.32

Supplemental Table S6: Acrophase results adding additional adjustment for a) days of shift and b) consecutive days prior compared to results from the primary mixed models

Acrophase results									
	primary analysis adjusted			additional adjustment for days of shift			additional adjustment for prior consecutive days		
	GMR	95% CI upper	95%CI lower	GM R	95%CI upper	95% CI lower	GMR	95%CI upper	95% CI lower
**aMT6s**	8.57	6.55	10.58	9.08	6.34	11.83	8.41	6.30	10.52
**Androgens**									
11-oxoandrosterone/11-oxoetiocholanolone	1.70	-1.81	5.21	1.67	-1.81	5.15	1.62	-2.19	5.43
Testosterone	6.83	0.34	13.32	9.39	-1.86	20.64	7.08	-0.02	14.18
Epitestosterone	7.54	3.09	12.00	6.90	1.69	12.11	5.78	2.31	9.25
Androstenedione	3.60	1.13	6.08	3.92	0.78	7.06	3.65	1.08	6.23
Androsterone	3.72	0.55	6.89	3.53	0.12	6.94	3.87	0.43	7.32
Etiocholanolone	3.18	0.88	5.48	2.76	0.55	4.96	3.30	0.79	5.82
7-cysteinyltestosterone	2.30	0.18	4.42	1.96	0.14	3.78	2.29	0.09	4.49
11-hydroxyandrosterone	1.23	-1.39	3.86	1.42	-1.84	4.69	1.00	-2.25	4.26
Lyase[c]	0.07	-5.73	5.87	-0.16	-7.88	7.55	-0.52	-7.63	6.59
17β-hydroxysteroid dehydrogenase (17βHSD)[c]	5.32	-1.28	11.92	2.72	-1.47	6.91	4.71	-1.40	10.82
5α-reductase[c]	-3.22	-8.51	2.06	-5.08	-14.40	4.23	-5.78	-15.07	3.50
Testosterone metabolism[c]	8.56	-0.33	17.44	5.98	-1.90	13.85	5.24	-0.22	10.70
**Progestogens**									
7-cysteinylprogesterone	0.55	-4.66	5.75	0.53	-2.09	3.16	0.58	-4.69	5.85
16-cysteinylprogesterone	3.03	0.93	5.13	3.88	0.65	7.11	3.27	0.90	5.64
17-hydroxyprogesterone	4.54	2.92	6.16	4.18	2.42	5.93	4.66	2.95	6.37
17-hydroxy-pregnanolone	2.19	0.33	4.05	2.13	-0.16	4.42	1.90	0.22	3.57
Pregnantriol	0.82	-1.49	3.13	0.69	-1.14	2.52	0.77	-1.74	3.27
**Estrogens**									
1-cysteinylandrostenedione	-2.66	-8.66	3.34	-3.20	-10.92	4.52	-3.33	-10.88	4.21
7-cysteinylandrostenedione	0.99	-3.74	5.71	0.75	-1.99	3.49	1.25	-2.75	5.24
Estrone	-1.50	-7.70	4.69	-2.21	-10.92	6.50	-3.16	-12.65	6.33
Aromatase[c]	-5.26	- 12.04	1.53	-4.16	-10.84	2.52	-5.74	-14.11	2.64

Supplemental Figure S1: Schematic of data collection and interval of time between the two points of data collection for all 44 participants

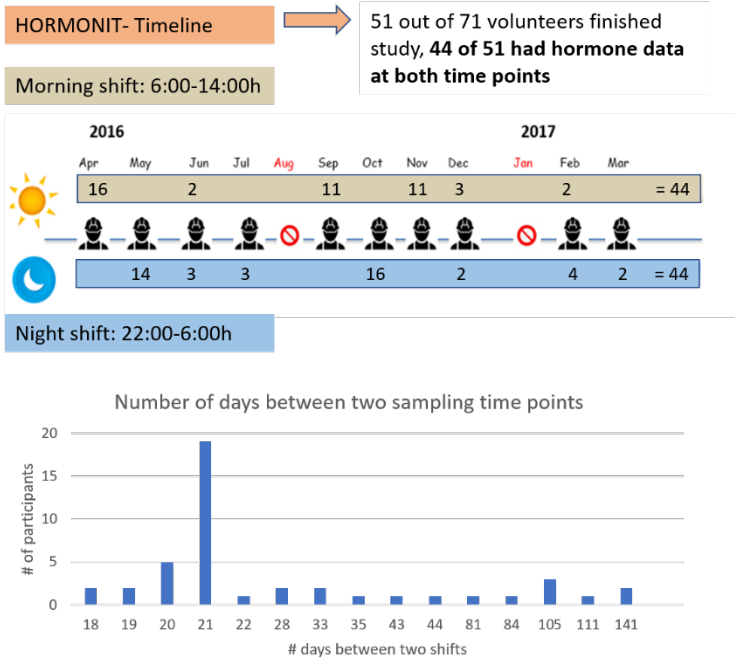

Supplemental Figure S2: Cosinor curves for overall production, acrophase, mesor and amplitude of aMT6s, testosterone and 16-cysteinyl progesterone among workers during the early morning day shift and night shift

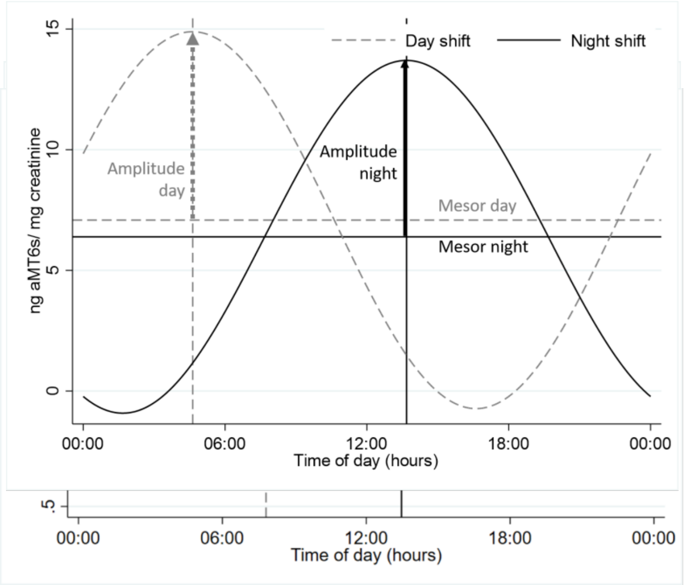

Supplement: Supplementary material [file SJWEH-48-41-S001.pdf]
